# Genome Sequence of *Bacillus* sp. Strain UMTAT18 Isolated from the Dinoflagellate *Alexandrium tamiyavanichii* Found in the Straits of Malacca

**DOI:** 10.1128/genomeA.01106-16

**Published:** 2016-10-06

**Authors:** Muhd Danish-Daniel, Gan Han Ming, Mohd Ezhar Mohd Noor, Yeong Yik Sung, Gires Usup

**Affiliations:** a School of Fisheries and Aquaculture Sciences, Universiti Malaysia Terengganu, Kuala Terengganu, Terengganu, Malaysia; b Institute of Marine Biotechnology, Universiti Malaysia Terengganu, Kuala Terengganu, Terengganu, Malaysia; c School of Science, Monash University Malaysia, Jalan Lagoon Selatan, Bandar Sunway, Petaling Jaya, Selangor, Malaysia; d Monash University Malaysia Genomics Facility, Jalan Lagoon Selatan, Bandar Sunway, Petaling Jaya, Selangor, Malaysia; e Faculty of Science and Technology, School of Environment and Natural Resource Sciences, Universiti Kebangsaan Malaysia, Bangi, Selangor, Malaysia

## Abstract

*Bacillus* sp. strain UMTAT18 was isolated from the harmful dinoflagellate *Alexandrium tamiyavanichii*. Its genome consists of 5,479,367 bp with 5,546 open reading frames, 102 tRNAs, and 29 rRNAs. Gene clusters for biosynthesis of nonribosomal peptides, bacteriocin, and lantipeptide were identified. It also contains siderophore and genes related to stress tolerance.

## GENOME ANNOUNCEMENT

Many *Bacillus* spp. have been reported to have biotechnological importance (1), while many others of this genus are also well-known pathogens (2). *Bacillus* spp. with the capacity to produce antimicrobial compounds and enzymes important to industry have been reported (3, 4). Some *Bacillus* spp. are used as probiotics in aquaculture to improve water quality, to enhance the hosts’ digestive systems, or to prevent the spread of diseases (5). *Bacillus* sp. strain UMTAT18 was isolated from the paralytic shellfish toxin-producing dinoflagellate *Alexandrium tamiyavanichii* found in the Straits of Malacca, Malaysia. We sequenced the genome of this strain to gain insights into its potential role as a probiotic for aquaculture applications*.*

*Bacillus* sp. strain UMTAT18 was cultured in marine broth 2216 (MB; Difco). Genomic DNA was then extracted using the GF-1 nucleic acid extraction kit (Vivantis, Malaysia). Sequencing was performed on the Illumina HiSeq2000 platform, generating 38,220,522 raw FASTQ paired-end reads. Two million reads were subsampled for error correction and *de novo* assembly using SPAdes version 3.1.0 (6). The resulting contigs were used for scaffolding, followed by gap-closing using SSPACE version 2.0 and GapFiller version 1.11 (7, 8). Sixty-nine gap-filled contigs with an *N*_50_ of 5 sequences longer than 317,317 bp were produced, and the total sequence length was 5,479,367 bp with a 73× coverage.

The Prokka version 1.8 annotation pipeline, comprised of Prodigal version 2.60, RNAmmer version 1.2, and Aragorn version 1.2.36, was used to annotate the genome, predicting 5,546 open reading frames, 29 rRNAs, and 102 tRNAs (9–12). The predicted 16S rRNA was queried with BLASTn (13) against the nucleotide collection database, identifying our sample as belonging to the genus *Bacillus*. Further validation of the species was performed using GGDC2.1 *in silico* genome-to-genome comparison of the UMTAT18 strain to other closely related *Bacillus* spp., showing that strain UMTAT18 is a novel species within the *Bacillus cereus* group (14). InterProScan5 was used to provide additional annotation to the predicted protein sequences (15). Furthermore, antiSMASH was used to identify the presence of secondary metabolite biosynthesis gene clusters in the genome (16).

*Bacillus* sp. UMTAT18 carries genes involved in the biosynthesis of bacillibactin and siderophore, which suggests that this strain could be applied in aquaculture to deprive pathogens that require iron for growth and pathogenesis (17). The genome of this strain also contains genes involved in the synthesis of antimicrobial properties like bacteriocin, lantipeptide, and nonribosomal peptide synthase, which allow it to compete with other bacteria in the aquatic environment. This strain possesses other environmental stress-adaptation-related genes, including those for osmotic and oxidative stress. The genome of strain UMTAT18 provides valuable information at the molecular level as a candidate probiotic for aquaculture applications.

### Accession number(s).

This whole-genome shotgun project has been deposited in DDBJ/EMBL/GenBank under the accession number JSFD00000000.
